# Synthesis and Characterization of a New Cu(II) Paddle-Wheel-like Complex with 4-Vinylbenzoate as an Inorganic Node for Metal–Organic Framework Material Design

**DOI:** 10.3390/ma16134866

**Published:** 2023-07-06

**Authors:** Egla Yareth Bivián-Castro, Marcos Flores-Alamo, Roberto Escudero, Virginia Gómez-Vidal, José J. N. Segoviano-Garfias, Jesus Castañeda-Contreras, Quetzalcoatl Enrique Saavedra-Arroyo

**Affiliations:** 1 Centro Universitario de los Lagos, Universidad de Guadalajara, Av. Enrique Díaz de León 1144, Col. Paseos de la Montaña, Lagos de Moreno 47460, Jalisco, Mexico; 2 Facultad de Química, Universidad Nacional Autónoma de México, Av. Universidad 3000, Circuito Exterior S/N, Coyoacán, Mexico City 04510, Mexico; 3 Instituto de Investigaciones en Materiales, Universidad Nacional Autónoma de México, Av. Universidad 3000, Circuito Exterior S/N, Coyoacán, Mexico City 04510, Mexico; 4 Instituto de Química, Universidad Nacional Autónoma de México, Av. Universidad 3000, Circuito Exterior S/N, Coyoacán, Mexico City 04510, Mexico; 5 División de Ciencias de la Vida, Carr. Irapuato-Silao Km. 12.5, Ex-Hacienda El Copal, Irapuato 36821, Guanajuato, Mexico; 6 Instituto Tecnológico Superior de Irapuato, Carr. Irapuato-Silao Km. 12.5, Ex-Hacienda El Copal, Irapuato 36821, Guanajuato, Mexico

**Keywords:** copper, paddle-wheel-like, 4-vinylbenzoic acid, open coordination sites, metal–organic frameworks

## Abstract

**Highlights:**

- A new Cu(II) paddle-wheel-like complex with vinylbenzoic acid was synthesized and characterized.
- The replacement of solvent molecules by water molecules from humid air at the open Cu^2+^ coordination sites of the complex should be explored further.

**Abstract:**

A new Cu(II) paddle-wheel-like complex with 4-vinylbenzoate was synthesized using acetonitrile as the solvent. The complex was characterized by X-ray crystal diffraction, FT-IR, diffuse reflectance spectroscopy, thermogravimetric, differential scanning calorimetric, magnetic susceptibility, and electronic paramagnetic resonance analyses. The X-ray crystal diffraction analysis indicated that each copper ion was bound at an equatorial position to four oxygen atoms from the carboxylate groups of the 4-vinylbenzoate ligand in a square-based pyramidal geometry. The distance between the copper ions was 2.640(9) Å. The acetonitrile molecules were coordinated at the axial position to the copper ions. Exposure of the Cu(II) complex to humid air promoted the gradual replacement of the coordinated acetonitrile by water molecules, but the complex structure integrity remained. The EPR spectra exhibited signals attributed to the presence of a mixture of the monomeric (S = ½) and dimeric (S = 1) copper species in a possible 3:1 ratio. The magnetic studies revealed a peak at 50–100 K, which could be associated with the oxygen absorption capacity of the Cu(II)–vba complex.

## 1. Introduction

Metal–organic frameworks (MOFs) are an interesting class of porous crystalline solids that are built by alternating the interconnection between inorganic nodes and organic linkers. MOF arrangement designs mostly derive from the inorganic nodes chosen as the building blocks; the available coordination sites at the metal centers are key to their application. Typically, Lewis base molecules can ligate and form weak coordination bonds with open coordination sites (OCSs). Their reversible-dissociable mechanism has been demonstrated to play an important role in the potential applications of MOFs, such as chemical separation, gas sorption, and catalysis. The activation process removes the coordinated molecules at the OCSs of the MOFs (the pre-coordinating solvent, the pore-filling guest, and the solvent molecules used during the synthesis); this essential step must be carried out before their use. HKUST-1 (HK), or Basolite, is an MOF built with multiple links between the Cu^2+^ paddle-wheel-like inorganic nodes and four 1,2,5-benzenetricarboxylate (BTC) linkers. HK-MOF is a good example of a material that possesses a high concentration of OCSs [1,2,3]. In nature, copper ions are particularly abundant in the Earth’s crust [4]; copper is an essential metal in several cofactors that are fundamental to numerous bioinorganic activities [5,6,7], such as oxygen transport [8], electron transfer [9], and enzyme activity [10]. In analogy to the catalytic cofactors found in nature in which the metal ion is always an essential part of the active species, numerous copper complexes have been synthetized for use as catalysts for the oxidation reactions of several substrates, such as alcohols and phenols. The interchange of ligands in copper complexes allows for the available coordination sites, with subsequent Lewis acid behavior of the metal center, to be ready for the incorporation of the substrate into catalytic reactions. Several copper systems have two or more copper ions at the active site, with functions that cannot be achieved by one copper ion alone. Thus, the synthesis of materials like MOFs with multiple copper ions in their structure is of scientific interest to further our understanding of certain biochemical mechanisms and for their potential use as functional materials [11,12,13]. In particular, Cu-MOFs are used for the adsorption of carbon dioxide, methane, and nitrogen [14]. Copper is an appropriate metal for the generation of paddle-wheel-like structures [15,16,17], which can be applied to biochemical processes, such as gas separation, adsorption, and catalysis [15]. Recently, we reported on the copper complex [Cuphen(vba)_2_H_2_O] (vba: 4-vinylbenzoic acid; phen: phenanthroline), from which a corresponding metal-imprinted polymer (MIP), [Cuphen(vba)_2_H_2_O-*co*-EGDMA]*_n_* (EGDMA: ethylene glycol dimethacrylate), was successfully obtained and used as an adsorbent for heavy metals [18]. 4-Vinylbenzoic acid is a styrene derivative that has been explored to obtain functional polymers with several applications [19]. Several analogous ligands of 4-vinylbenzoic acid have been used to obtain metal–organic frameworks [20]. In this paper, we present a novel Cu(II) paddle-wheel-like complex for use as an inorganic node to continue our research into the preparation of new materials, such as MOFs. Different organic linkers, like BTC, were tested to build interesting MOF structures. The synthesis and thermal behavior of bisacetonitriletetravinylbenzoato-bridged copper(II) were examined. The X-ray crystal structure of the complex showed a square-channel arrangement of [Cu_2_(µ-vba)_4_(CH_3_CN)_2_], in which two copper metal centers were bonded to four 4-vinylbenzoate ligands and two acetonitrile (MeCN) solvent molecules. Gradually, the solvent molecules were replaced by environmental water molecules, identified by spectroscopic characterization. The magnetic susceptibility results demonstrated the oxygen adsorption capacity of the complex.

## 2. Materials and Methods

### 2.1. Chemicals

The triethylamine, 4-vinylbenzoic acid (vba), acetonitrile (MeCN), and dimethylformamide (DMF) were provided by Sigma-Aldrich (St. Louis, MI, USA). The Cu(NO_3_)_2_·2.5 H_2_O was provided by Fermont (Monterrey, Mexico). All reagents were of an analytical grade and were used without further purification.

### 2.2. Apparatus

The molar conductivity was measured using Conductronic PC45 equipment (Conductronic, Puebla, México), with a fresh 1 mM DMF solution. FT-IR spectra were obtained using a Perkin-Elmer Spectrum RXI spectrometer (Perkin-Elmer, Waltham, MA, USA) from a KBr pellet ranging from 4000 to 400 cm^−1^. The solid sample was measured by diffuse reflectance from 200 to 900 nm on an Ocean Optics QE65000 spectrometer, with an Ocean Optics ISP-30R integrating sphere. Variable temperature and magnetic measurements of the polycrystalline samples were carried out using a Quantum Design SQUID magnetometer (Quantum Design North America, San Diego, CA, USA). The temperature was varied between 4 and 300 K, according to a zero-field-cooling (ZFC)/field-cooling (FC) procedure at 1000 Oe. The data were corrected for diamagnetism contributions with Pascal’s constants [21]. Electron paramagnetic resonance spectra were obtained from the polycrystalline samples in quartz tubes at room temperature and 77 K, with a Jeol JES-TE300 spectrometer (JEOL USA, Inc., Peabody, MA, USA) operating at an X-band frequency (near 9.4 GHz) with a 100 kHz field modulation and a cylindrical cavity (TE_011_ mode). The external measurement of the static magnetic field was obtained using a Jeol ES-FC5 precision gaussmeter [22]. Thermogravimetric analyses and differential calorimetric scanning were carried out using TA Instruments Q600 equipment (with a heating rate of 5 °C/min), with a nitrogen flux of 20 cm^3^/min. XRPD patterns were collected using an Empyrean diffractometer (Panalytical Ltd., Malvern, UK), using CuKα radiation (λ = 0.15418 nm).

### 2.3. Synthesis of the Cu(II) Paddle-Wheel Complex

In 20 mL of acetonitrile, 0.6 mmol (0.1005 g) 4-vinylbenzoic acid and 1.8 mmol (0.417 g) Cu(NO_3_)_2_·2.5 H_2_O were completely mixed; 139 µL triethylamine was then added. The mixture was stirred for 1 h at room temperature. The resultant green solution was filtered to avoid any impurities and left in refrigeration. After two weeks, deep green prism crystals of the coordination complex [Cu_2_(µ-vba)_4_(CH_3_CN)_2_] had formed. The yield was 0.042 g (35.2%). The crystals could be solubilized in dimethylformamide.

### 2.4. Crystallography of the Cu(II) Paddle-Wheel Complex [Cu_2_(µ-vba)_4_(CH_3_CN)_2_]

A suitable single crystal of the Cu(II) complex [Cu_2_(µ-vba)_4_(CH_3_CN)_2_] was mounted on glass fiber under a cryogenic system. The crystallographic data were collected using an Oxford Diffraction Gemini Atlas diffractometer (Oxford Diffraction Ltd., Abingdon, UK) with a CCD area detector (λ_MoKα_ = 0.71073 Å) and a monochromator of graphite at 130 K. The CrysAlisPro and CrysAlis RED software packages v1.171.36.32 (Oxford Diffraction Ltd., Abingdon, UK) were used for the data collection and integration [23]. The double-pass method of scanning was used to exclude any noise. The collected frames were integrated using an orientation matrix determined from the narrow-frame scans. The cell constants were determined by global refinement. A numeric absorption correction [24] was applied. The structure solution and refinement were carried out using the SHELXS-2018 [25] and SHELXL-2018 [26] programs (Institute of Inorganic Chemistry, Göttingen, Germany). All the non-hydrogen atoms were anisotropically refined. H atoms attached to C atoms were placed in geometrically idealized positions and refined to ride on their parent atoms, with C—H = 0.95–0.98 Å and Uiso (H) = 1.2 Ueq (C) for the aromatic methine and methylene groups, and Uiso (H) = 1.5 Ueq (C) for the methyl groups. The drawing of the molecular structure was performed using Mercury CSD software v2023.1.0 [27]. The crystal data and structure refinement for [Cu_2_(µ-vba)_4_(CH_3_CN)_2_] are shown in Table 1. The corresponding selected bond lengths (Å) and bond angles (°) for the complex are shown in Table S1. The crystallographic data were deposited at the Cambridge Crystallographic Data Center (Supplementary Material, CCDC: 1551224).

## 3. Results and Discussion

### 3.1. Crystal Structure of the Cu(II) Paddle-Wheel Complex [Cu_2_(µ-vba)_4_(CH_3_CN)_2_]

**Figure 1:**
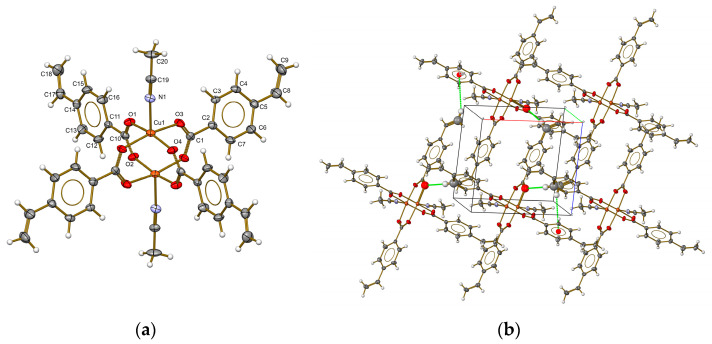
ORTEP diagram of the discrete unit (**a**) and crystal network along the *a*–*c* plane showing the intermolecular contacts (**b**) of the copper complex [Cu_2_(µ-vba)_4_(CH_3_CN)_2_]. Ellipsoids of 50% probability.

**Figure 2:**
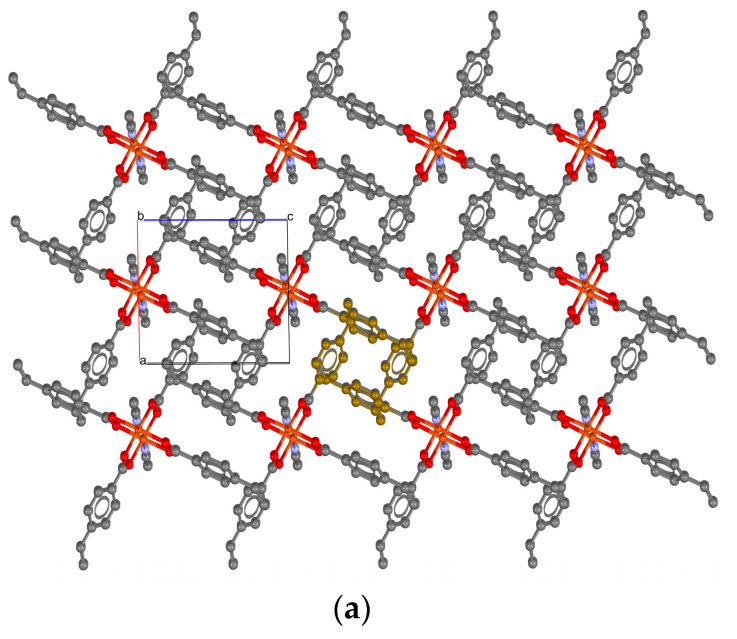

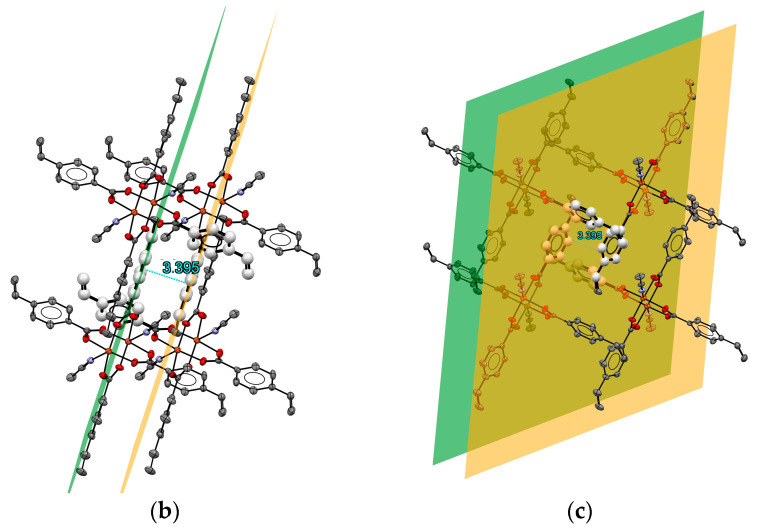
The 2D herringbone architecture (**a**), as well as 1D (**b**) and 2D (**c**) square-channel views of the copper complex [Cu_2_(µ-vba)_4_(CH_3_CN)_2_].

Single deep green crystals suitable for the X-ray measurements of the copper complex were obtained. A discrete unit of [Cu_2_(µ-vba)_4_(CH_3_CN)_2_] with the coordination of the metal and atomic labeling is shown in Figure 1a. Each copper(II) ion had a square pyramidal geometry. The base consisted of four oxygen atoms from the carboxylate groups of each of the four vba ligands and an expected Cu—O bond distance of 1.954(2) to 1.966(2) Å. The apical position of the pyramid was occupied by a nitrogen atom from the MeCN ligand with a Cu—N bond distance of 2.239(3) Å. The tau descriptor for five coordinations was expressed as the difference between the angles of the bonds O(2)—Cu—O(1) and O(3)—Cu—O(4). This, divided by 60, resulted in a value of τ = 0.003, which is close to the ideal value of τ = 0 for a square pyramid [28]. The Cu⋯Cu separation of 2.640(9) Å is close to that in other compounds with similar structures [29,30,31]; analogous interatomic copper distances can be found in several laccase active sites [32]. Copper(II) arylcarboxylates are generally complexes with two close proximity Cu(II) ions surrounded by bidentate ligands, such as vba, giving rise to a paddle-wheel-like structure. As with others, the cage structure of the copper(II) vinylbenzoate, shown in Figure 1a, was expected to be stronger and highly stable due to the presence of high electron density at the bridging groups supplied by the phenyl rings, making stronger Cu—O coordinated bonds [29]. In the crystal network (Figure 1b), there were intermolecular interactions of C—H…O of a hydrogen bond-type and C—H…π. The intermolecular contact C(18)—H(18)…Cg(1) from the benzene ring centroid was located at 3.5424(5) Å. The hydrogen bond types C(4)—H(4)…O(1) (2.4955(2) Å) and C(17)—H(17)…O(3) (2.6458(3) Å) were related to the symmetry operations 2 − x, 1 − y, −z and x, −1 + y, and 1 + z, respectively. All intermolecular interactions were observed along the a–c plane and led to an infinite laminar array in the supramolecular network. The 2D herringbone architecture of the Cu(II)-complex crystal packing is shown in Figure 2a. Detailed views of the square channels are shown in Figure 2b,c, with an approximate dimension of 3.4 Å; this was formed through the arrangement of the vba ligands.

### 3.2. Synthesis and Characterization

**Figure 3:**
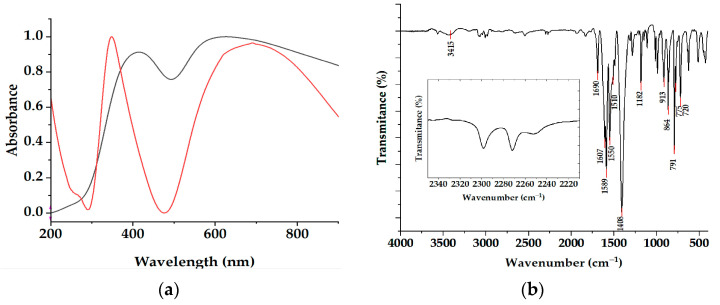
Diffuse reflectance spectra of the fresh sample (black line) and the sample exposed to environmental moisture (red line) (**a**). Mid-infrared (**b**) spectra of the Cu(II) paddle-wheel complex.

The synthesis of the copper paddle-wheel complex in acetonitrile resulted in the two solvent molecules being coordinated at the metal center; this was confirmed by single X-ray diffraction. After the crystals were immediately redissolved in a dimethylformamide solution, the molar conductivity measurement was Λ_M_ 6.0 Ω^−1^ cm^2^ mol^−1^, as expected for a non-electrolyte compound in which the ligands were coordinated with the metal ion [33]. To identify the electronic transition bands of the copper complex, the solid-state diffuse reflectance spectra was analyzed. Two main reflectance bands were observed. A comparison between the diffuse reflectance spectra of a fresh sample against others exposed to humidity is shown in Figure 3a (black and red lines, respectively). An evident shift in the bands was observed. The maximum absorption at 414.7 nm in the black spectrum was displaced to 348.4 nm in the red one; either of the observed signals could be attributed to ligand–metal charge transitions. These electronic transitions have previously been identified in several polymeric copper carboxylic compounds. In the visible region, the maximum absorption at 626.9 nm in the black spectrum shifted to 694.4 nm in the red one. These electronic transitions could be attributed to the d–d transitions of copper(II) complexes with a square-based pyramidal coordination environment [34,35]. The green color of the Cu(II)-complex crystals was conserved, provided that they remained in their mother liquor of MeCN. When the crystals were isolated and exposed to environmental moisture, their color gradually turned pale blue. The color changes and the observed band displacements in the reflectance spectra, as shown in Figure 3a, could be associated with the ability of the Cu(II) paddle wheel to replace the pre-coordinated MeCN solvent by other Lewis-base polar guest molecules, as has been previously reported for other Cu(II) paddle-wheel-like structures [1,36]. The dynamic decoordination–coordination behavior of the acetonitrile-promoted copper sites was observed with the possibility of acting as Lewis acids; therefore, a catalytic property of the material could be speculated. Several commercial materials, such as the known HKUST-1 or Basolite, along with the interesting topology of the Cu(II) paddle wheel, can be used as important catalyst materials [37]. Therefore, we posited that sufficiently fed H_2_O molecules with environmental moisture could be coordinated to the metal center of a paddle wheel whose structure was preserved. The mid-infrared spectrum shown in Figure 3b supports the characterization of the copper complex structural integrity. The expected signals due to the ligand and solvent molecules were identified.

The spectrum showed a broad band at 3438 cm^−1^, which was assigned to the O—H stretching vibrations of lattice water molecules. A broad band between 1588 cm^−1^ and 1404 cm^−1^ was split and included the asymmetric *ν*_asym_(COO^−^) and symmetric *ν*_sym_(COO^−^); these were present because of the interactions by the 4-carboxylic groups of the vba ligands with the metallic ion. The value of Δ*ν*, which is the difference between the *ν*_asym_(COO^−^) and symmetric *ν*_sym_(COO^−^), was 184 cm^−1^. This was indicative of the coordination mode from the COO^−^ to the copper ions [38]. This result could also indicate the symmetrical bridging coordination mode of the carboxylate (syn; syn-η^1^:η^1^:μ^2^), giving rise to a paddle-wheel-type structure. At 1694 cm^−1^, the C=C signal from the vinyl group of vba ligands was found. A sharp signal at 1543 cm^−1^ was assigned to the aromatic double C=C bonds. The signal of the Cu—O vibrations was located at 718 cm^−1^ [39,40,41]. The inset in Figure 3b allowed us to identify two main signals at 2298 and 2272 cm^−1^ that were assigned to the C≡N vibration modes of the MeCN. Comparing our results with those of the systematic studies by [2], only two signals were identified after the elimination of the Cu^2+^-coordinated MeCN by an exchange with H_2_O. These were from 2350 to 2200 cm^−1^, in the region assigned to the presence of the [Cu(MeCN)_4_]^+^ complex. The mechanism for the formation of the Cu^+^ complex was proposed as a “ship-in-a-bottle” model, according to which the Cu^+^ and MeCN components were post-assembled in the small cage in the paddle-wheel complex after they had penetrated the cage [2]. As shown in the inset of Figure 3b, we noticed a diminishing signal at 2252 cm^−1^, which corresponded with only one C≡N vibration mode for the coordinated MeCN at the inorganic node of [Cu_2_(µ-vba)_4_(CH_3_CN)_2_].

**Figure 4:**
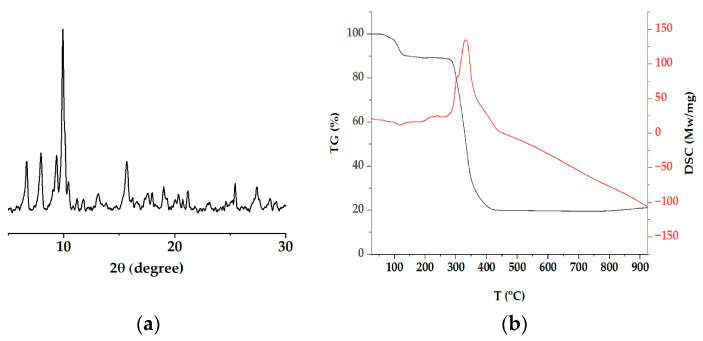
Powder X-ray diffractogram (**a**) and thermal analyses curves (TGA and DSC, black and red lines, respectively) (**b**) for the copper complex.

Figure 4a shows the PXRD pattern of the Cu(II) paddle wheel, demonstrating the polycrystalline structure of the aggregates with five main peaks (2*θ* = 6.63°, 7.92°, 9.32°, 9.94°, and 15.65°).

The particle size (D, in nm) of the copper complex was found to be 23.32 nm, which was calculated using the Scherrer equation [42,43]. The results on the spacing between the diffraction planes (d) for the main peaks in the complex diffractogram are presented in Table 2. The last calculations were performed using Bragg’s law.

The PXRD also showed that the structural integrity of the complex was conserved, even though the solvent coordination was removed. The PXRD pattern for the Cu(II) complex had almost the same pattern as those previously reported with similar paddle-wheel conformations, such as copper(II) fluorobenzoate, HKUST-1, and MeCN-HK [1,2,29,43]. To investigate the thermal stability of the copper complex and the mobility of the solvent molecules within the complex, a TGA was conducted. Figure 4b shows the thermogravimetric curves, demonstrating the continuous weight loss below 280 °C that corresponded with the solvent molecules of water and acetonitrile (found 13%, calc. 13.76%). The paddle-wheel arrangement started to thermally decompose at approximately 300 °C. The last loss of mass weight, around 20%, was attributed to metal residue. The DSC showed endothermic and exothermic peaks, which indicated the steps of complex decomposition. The peak at 121 °C was due to the loss of water and acetonitrile solvent molecules. The endothermic peak corresponded with the loss of coordinated water molecules. The exothermic peak at 331 °C provided evidence of the thermal degradation of the vba ligands with the corresponding destabilization of the paddle-wheel arrangement. This behavior has previously been observed in several paddle-wheel compounds [37,38,39,44].

### 3.3. Magnetic and Electronic Paramagnetic Resonance Results for the Copper Complex

#### 3.3.1. Magnetic Behavior

**Figure 5:**
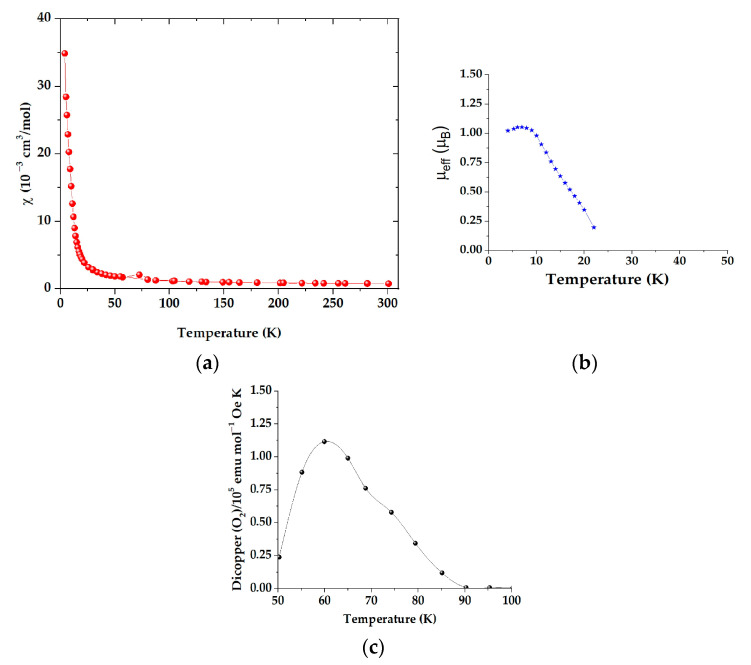
Magnetic susceptibility (**a**,**b**) moment curves for the copper complex; (**c**) oxygen adsorption of the complex (red and black circles, as well as blue stars are the data collected during each of the experiments).

The magnetic properties of the copper complex as a function of temperature are shown in Figure 5. The magnetic susceptibility curve in Figure 5a exhibits a behavior characteristic of antiferromagnetically coupled copper(II) pairs. As shown in Figure 5b, the magnetic moment reached a *µ_eff_* of 1.05 B.M. at 7 K. The coupling interaction between unpaired electrons in dimeric paddle-wheel structural systems has been shown to occur through the super-exchange pathway, rather than through direct interactions between the two central copper atoms, with a binuclear oxygen-bridged structure to vba ligands [45]. Figure 5c shows a detected peak from 50 to 100 K, which was associated with the oxygen absorption by the structure of the Cu(II) paddle wheel [45]. At room temperature, the corrected molar magnetic susceptibility *χ_M_^corr^* was 7.689 × 10^−4^ cm^3^/mol; when corrected for diamagnetism, it was −405.9 × 10^−6^ cm^3^/mol [21]. The complex had a magnetic moment *µ_eff_* of 1.36 B.M. at 300 K. This value corresponds well with those of similar complexes, previously reported [31].

#### 3.3.2. Electronic Paramagnetic Resonance Results

**Figure 6:**
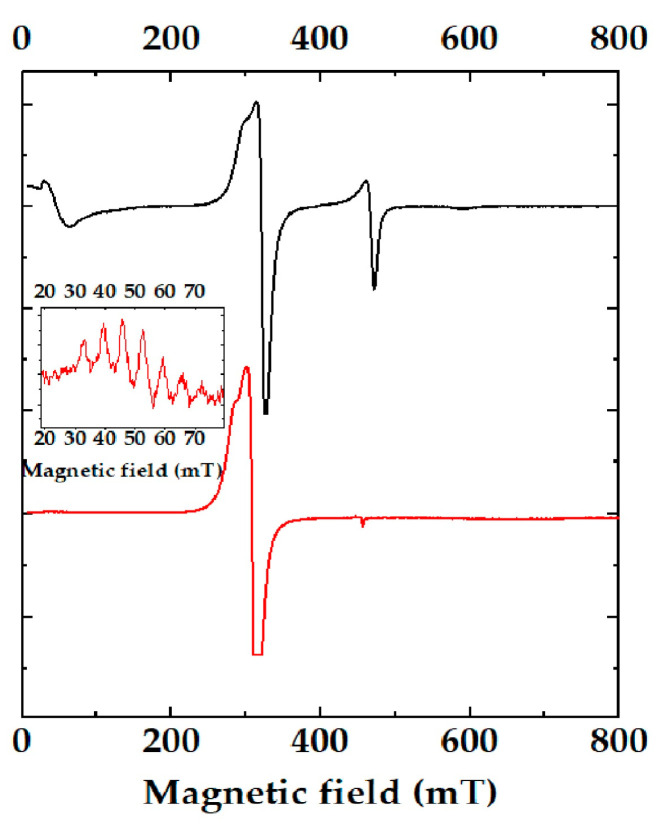
Electronic paramagnetic resonance spectra of the copper complex at room temperature (black line) and in a solid state at 77 K (red line). The inset corresponds with the pattern of seven hyperfine lines.

The electronic paramagnetic resonance (EPR) spectra of the complex as a function of temperature is shown in Figure 6. The signals observed in the EPR spectra could be attributed to the presence of a mixture between the monomeric (S = ½) and the dimeric (S = 1) copper species, in a possible 3:1 ratio. The spectra corresponding with the monomer appeared at the center field as an axial signal without hyperfine coupling, with *g*_⊥_ = 2.107. In a smaller proportion, a broad feature was observed that was attributed to a well-resolved triplet state (S = 1). The spectrum was axially symmetrical, and perpendicular components (Z) appeared at the extreme ends of the spectrum. Relatively high D values appeared and could be analyzed with the spin Hamiltonian of D ≠ 0 and E ≈ 0 as *H* = *βHg S* + *D [S_z_*^2^ *− 2/3] + [S_x_*^2^ *− S_y_*^2^*]E*. The spin Hamiltonian parameters calculated were *H_z_*_1_ = 45.941 mT, *H_z_*_2_ = 590.082 mT, *H_⊥_*_2_ = 472.11 mT, *g_‖_* = 2.11, and *D* = 0.287 cm^−1^. The calculated parameters agreed well with those previously reported for analogous systems [46]. The parameters were calculated taking into account the equations proposed by Wasserman et al. [47]; namely, *H_z_*_1_ = (*g_e_*/*g_‖_*)(*H*_0_ − *D′*) and *H_z_*_2_ = (*g_e_*/*g_‖_*)(*H_0_* + *D′*), where *D′* = *D*/*g_e_ β*, *g_e_* = 2.0023, and *H*_0_ = *hν*/*g_e_ β*. The experimental *D* value calculated was the sum of two contributions to the observed zero-field splitting of the spin triplet. One was due to the dipole–dipole interaction between the unpaired spins in the two copper atoms. When the temperature was lower than 77 K, the signals became sharp and their intensity significantly decreased; almost exclusively, signals corresponding with the diluted paramagnetic centers S = ½ were observed [22]. The inset in Figure 6 shows the appearance of a pattern of seven hyperfine lines in *H_z_*_1_ that confirmed the coupling of two Cu^2+^ S = ½. The calculated parameters were *g_‖_* = 2.2789 and *g*_⊥_ = 2.1077.

## 4. Conclusions

A new copper(II) paddle-wheel-like complex was synthetized and characterized. The crystal structure of the complex [Cu_2_(μ-vba)_4_(CH_3_CN)_2_] showed a complex with two Cu(II) ions, each pentacoordinated with a square pyramidal geometry. The copper ions were coordinated to four vba ligands and to two solvent molecules of acetonitrile at axial positions. A 2D herringbone architecture, with several square channels formed through the vba ligands, was arranged by the complex. The exposure of the complex to environmental moisture promoted the gradual replacement of the acetonitrile ligands with water molecules, even though the structural integrity of the paddle wheel was preserved; this was in agreement with the presented characterization. The existence of a mixture of two chemical species was identified in the electronic paramagnetic studies. These corresponded with the Cu(II)–vba paddle wheel synthetized in this research and the suggested post-assembled [Cu(MeCN)_4_]^+^ complex that could be formed when Cu^2+^-coordinated MeCN was exchanged with H_2_O. Magnetic susceptibility studies proved the capacity of the copper complex for oxygen catchment. Further detailed studies are required to explore the effect of the exposure time of the complex crystals to humid air, and to discover the conditions required to enhance the hydrolytic stability of the metal center. These could, significantly, be related to the stability of the synthesis of future MOFs from this inorganic node.

## Figures and Tables

**Table 1:** Crystal data and structure refinement for the Cu(II) paddle-wheel complex [Cu_2_(µ-vba)_4_(CH_3_CN)_2_].

Empirical Formula	C_40_H_34_Cu_2_N_2_O_8_
Formula weight	797.79
Crystal system	Triclinic
Space group	P-1
Unit cell dimensions	
*a*	9.7660(9) Å
*b*	10.2317(10) Å
*c*	10.8093(12) Å
α	63.036(11)°
β	80.967(9)°
γ	71.534(9)°
*V*	913.00(16) Å^3^
Z	1
Density (calculated)	1.451 Mg/m^3^
Absorption coefficient	1.220 mm^−1^
*T*	130(2) K
*F* (000)	410
Wavelength	0.71073 Å
*θ* range	3.62 to 26.05°
Index ranges	−11 ≤ *h* ≤ 12; −10 ≤ *k* ≤ 12; −10 ≤ *l* ≤ 13
Collected reflections	6691
Independent reflections	3610 [*R*_int_ = 0.0497]
Completeness to theta = 26.05°	99.8%
Refinement method	Full-matrix least-squares on *F*^2^
Data/restraints/parameters	3610/0/236
Goodness-of-fit on *F*^2^	1.038
Final *R* indices [I > 2⌠(*I*)]	*R*_1_ = 0.0543; *wR*_2_ = 0.0848
*R* indices (all data)	*R*_1_ = 0.0857; *wR*_2_ = 0.1006
Largest diff. peak and hole	0.399/−0.436 e.Å^−3^

**Table 2:** PXRD data for the copper complex.

2θ (°)	D (nm)	d (Å)
6.633 ± 0.004	24.97	13.32
7.920 ± 0.003	22.91	11.16
9.326 ± 0.004	24.07	9.48
9.948 ± 0.001	22.10	8.89
15.659 ± 0.004	22.54	5.65

## Data Availability

Data are available from the corresponding author upon reasonable request.

## Supplementary Materials

The following supporting information can be downloaded at: https://www.mdpi.com/article/10.3390/ma16134866/s1, Figure S1: Electronic spectra in the UV region as a suspension in nujol, and in the visible region using DMF as solvent for the Cu(II) complex [Cu_2_(μ-vba)_4_(CH_3_CN)_2_]; Table S1: Selected bond distances and angles for the copper paddle-wheel complex, [Cu_2_(μ-vba)_4_(CH_3_CN)_2_].
