# Supplementary material for: Synthesis and Characterization of a New Cu(II) Paddle-Wheel-like Complex with 4-Vinylbenzoate as an Inorganic Node for Metal–Organic Framework Material Design
Source: Materials (Basel). 2023 Jul 6;16(13):4866. doi: 10.3390/ma16134866 (PMC10343663; doi:10.3390/ma16134866)
Supplement: Supplementary file 1 [file materials-16-04866-s001.zip › materials-2434231-supplementary.pdf]

Table S1. Selected bond distances (Å), and angles (°) for the copper paddle-wheel complex [Cu_2(μ-vba)$_4$(CH_3CN)$_2$].

Bond Length (Å)		Bond Angles (°)	
Cu(1) —O(1)	1.966(2)	O(1) —Cu(1) —O(4)	89.02(11)
Cu(1)—O(2)	1.965(2)	O(1) —Cu(1) —N(1)	98.29(11)
Cu(1)—O(3)	1.954(2)	O(1) —Cu(1) —O(2)	168.46(10)
Cu(1) —O(4)	1.964(2)	O(1) —Cu(1) —O(3)	88.88(11)
Cu(1)—O(3)	1.954(2)	O(4) —Cu(1) —N(1)	97.60(11)
Cu(1)—O(2)	1.965(2)	O(4) —Cu(1) —O(2)	89.99(11)
Cu(1) —N(1)	2.239(3)	O(4) —Cu(1) —O(3)	168.28(10)
Cu(1) —Cu(1)	2.640(9)	N(1) —Cu(1) —O(2)	93.24(10)
O(1) —C(10)	1.266(4)	N(1) —Cu(1) —O(3)	94.11(11)
O(2) —C(10)	1.260(4)	O(2) —Cu(1) —O(3)	89.76(11)
N(1) —C(19)	1.132(4)	Cu(1) —N(1) —C(19)	170.7(3)
C(19) —C(20)	1.460(5)	N(1) —C(19) —C(20)	178.6(4)
C(1) —C(2)	1.495(5)	O(3) —C(1) —O(4)	124.9(3)
C(8) —C(9)	1.315(6)	O(3) —C(1) —C(2)	116.8(3)

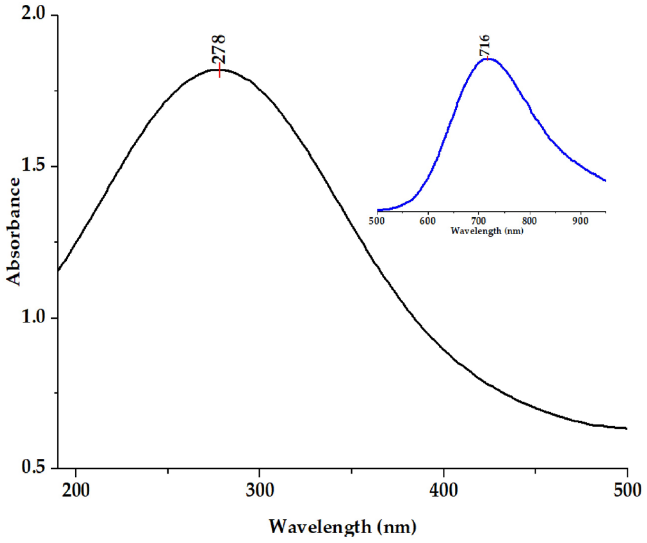

Figure S1. Electronic spectra in the UV region as a suspension in nujol, and in the visible region (inset) using DMF as solvent for the Cu(II) complex, [Cu_2(μ-vba)$_4$(CH_3CN)$_2$].
